# Multifunctionality of Rapeseed Meal Protein Isolates Prepared by Sequential Isoelectric Precipitation

**DOI:** 10.3390/foods11040541

**Published:** 2022-02-14

**Authors:** Radoslav Georgiev, Hristo Kalaydzhiev, Petya Ivanova, Cristina L. M. Silva, Vesela I. Chalova

**Affiliations:** 1 Department of Biochemistry and Molecular Biology, University of Food Technologies, 26 Maritsa Blvd, 4002 Plovdiv, Bulgaria; racho95@abv.bg (R.G.); petia_ivanova_georgieva@abv.bg (P.I.); 2 Department of Analytical Chemistry and Physicochemistry, University of Food Technologies, 26 Maritsa Blvd, 4002 Plovdiv, Bulgaria; hristo.kalaydzhiev@yahoo.com; 3 CBQF-Centro de Biotecnologia e Química Fina–Laboratório Associado, Escola Superior de Biotecnologia, Universidade Católica Portuguesa, Rua Diogo Botelho 1327, 4169-005 Porto, Portugal; clsilva@porto.ucp.pt

**Keywords:** rapeseed, sequential isoelectric precipitation, multifunctionality, protein isolates, antioxidant capacity

## Abstract

Rapeseed meal is a by-product of the oil-producing industry with a currently underestimated application. Two protein isolates, PI_2.5–8.5_ or PI_10.5–2.5_, were obtained from industrial rapeseed meal after treatment with an aqueous ethanol solution. The alkaline-extracted proteins were sequentially precipitated by two different modes, from pH 10.5 to 2.5, and vice versa, from 2.5 to 8.5, with a step of 1 pH unit. The preparation approach influenced both the functional and antioxidant properties of the isolates. The PI_10.5–2.5_ exhibited higher water and oil absorption capacities than PI_2.5–8.5_, reaching 2.68 g H_2_O/g sample and 2.36 g oil/g sample, respectively. The emulsion stability of the PI_2.5–8.5_, evaluated after heating at 80 °C, was either 100% or close to 100% for all pH values studied (from 2 to 10), except for pH 6 where it reached 93.87%. For the PI_10.5–2.5_, decreases in the emulsion stability were observed at pH 8 (85.71%) and pH 10 (53.15%). In the entire concentration range, the PI_10.5–2.5_ exhibited a higher scavenging ability on 2,2-diphenyl-1-picryl hydrazyl (DPPH) and hydroxyl radicals than PI_2.5–8.5_ as evaluated by DPPH and 2-deoxyribose assays, respectively. At the highest concentration studied, 1.0%, the neutralization of DPPH radicals by PI_10.5–2_ reached half of that exhibited by synthetic antioxidant butylhydroxytoluene (82.65%). At the same concentration, the inhibition of hydroxyl radicals by PI_10.5–2_ (71.25%) was close to that achieved by mannitol (75.62%), which was used as a positive control. Established antioxidant capacities add value to the protein isolates that can thus be used as both emulsifiers and antioxidants.

## 1. Introduction

Rapeseed oil is a valuable commodity widely used in the food industry [1] or other industrial sectors to produce biofuel, paper, textile, plastics, lubricants, and surfactants [2,3]. It is also used in agriculture for dust masking in swine barns [4] or as a biopesticide [5]. The enhanced demand for this vegetable oil worldwide results in high amounts of rapeseed meal as a by-product, which reached 38.8 million tons in 2018 [6] and 40 million tons in 2020 [7].

Currently, rapeseed meal has an underestimated application. Mainly, it is used as an inexpensive protein-rich ingredient in feed formulation [8,9]. However, the presence of substantial amounts of glucosinolates, tannins, and fibres limits the inclusion of the meal because of their negative effect on animal growth and physiology [10].

Alternatively, rapeseed meal could be used as a source for preparing protein isolates/concentrates. Recently, there has been an increasing interest in plant proteins. The global demand for protein is driven by various factors such as population growth, increases in urbanization and ageing, and altered customers’ preferences which are recently directed to a vegetable type of foods as a prerequisite for a healthy style of living [11]. In addition, the production of animal-based foods is associated with higher levels of greenhouse gases than plant-based foods, which reflects climate change [12,13].

Rapeseed protein isolates are “novel foods” introduced to the human diet [14]. However, they possess desired nutritional and functional characteristics that are highly dependent on raw material characteristics, seed processing, and protein isolate preparation mode [15,16]. Most protein isolates are prepared by isoelectric precipitation in the acidic pH area, where they exhibit the lowest solubility [17,18,19,20]. A low solubility limits protein isolate functionality and subsequent application. Kalaydzhiev et al. [21] used a different approach, i.e., sequential isoelectric precipitation, and obtained two protein isolates from industrial rapeseed meal with enhanced solubility. Protein solubility is a crucial determinant of remaining functional properties, and plant proteins with such a characteristic would be a value added to the food, agricultural, and pharmaceutical industries. These two protein isolates also contain phenols that could contribute to their functionality and overall benefits, although increasing their impurity. The protein isolates functional properties combined with the phenols antioxidant capacity may turn them into bioactive ingredients with multi-functional features [22,23].

The current study continues the previous one [21] aiming to evaluate the potential applicability of the newly obtained rapeseed meal protein isolates as multifunctional ingredients. For this purpose, water and oil absorption capacity and emulsifying properties at two boundary concentrations of NaCl (0.03 and 0.25 M) in a wide pH range (2.0–10.0), were determined. Antioxidant capacities were estimated by three methods differing by their principles, namely 2,2-diphenyl-1-picryl hydrazyl (DPPH), ferric reducing antioxidant power (FRAP), and 2-deoxy-D-ribose assays to better reveal the practical value of the isolates.

## 2. Materials and Methods

### 2.1. Materials

Rapeseed meal was manufactured and kindly donated by a local processing factory (OLIVA AD, Polski Trambesh, Bulgaria). All analytical grade reagents were from Merck KGaA (Darmstadt, Germany) via Fillab (Plovdiv, Bulgaria), and water used in all analyses was distilled.

### 2.2. Preparation of Protein Isolates

**Figure 1:**
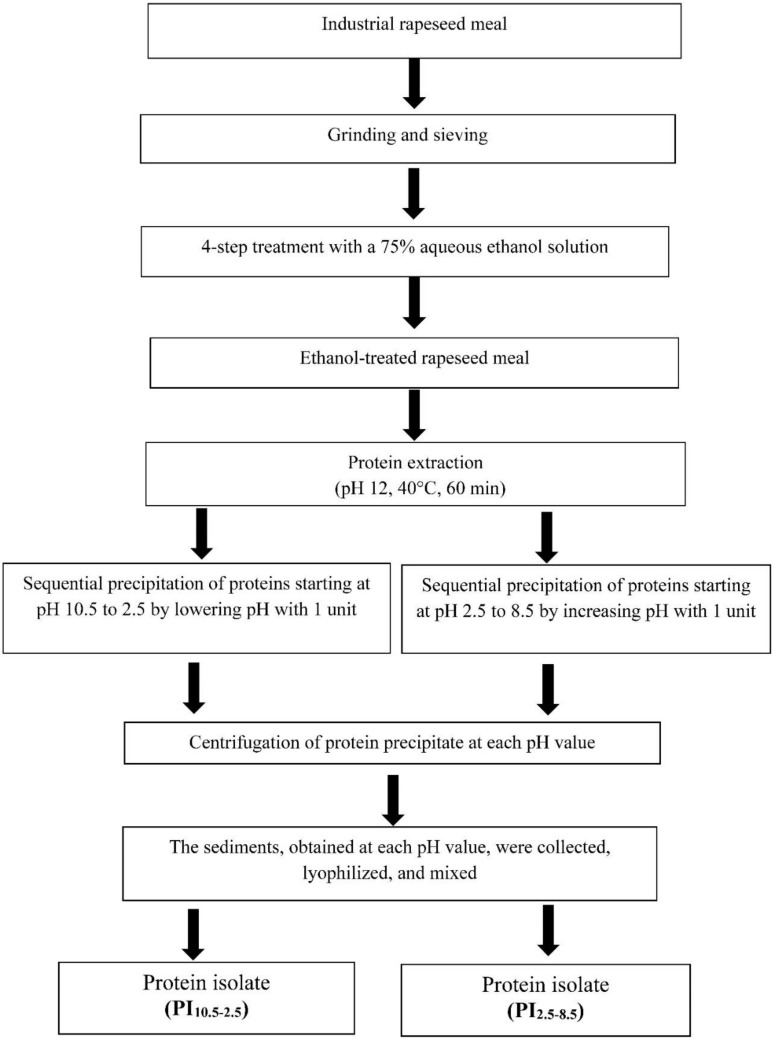
Preparation of protein isolates, PI_2.5–8.5_ and PI_10.5–2.5_, from ethanol-treated rapeseed meal.

Protein isolates were prepared from industrially manufactured rapeseed meal as previously described [21]. Briefly, the meal was ground and sieved. Uniformly sized particles (≤0.315 mm) were subjected to a 4-fold treatment with a 75% aqueous ethanol solution (*v*/*v*). The ethanol-treated rapeseed meal was further used to extract proteins at pH 12 (40 °C, 60 min). Two protein isolates were prepared by a vice versa sequence of precipitation at multiple pH values. The protein isolate PI_10.5–2.5_ was obtained by sequential precipitation of the proteins from the extract starting at pH 10.5 to 2.5 by lowering the pH by one unit. The second one, PI_2.5–8.5_, was obtained after a sharp decrease in extract pH to 2.5, followed by an increase in the pH to 8.5 with one unit. The pH was adjusted by using either NaOH or HCl as needed. The precipitates, obtained at each pH value, were collected by centrifugation (6000 rpm), lyophilized (Lyovac GT2, Leybold-Heraeus, Köln, Germany), and mixed to prepare the powdery protein isolates, PI_2.5–8.5_ or PI_10.5–2.5_. The procedure is schematically outlined in Figure 1.

### 2.3. Techno-Functional Properties of PI_2.5–8.5_ and PI_10.5–2.5_

#### 2.3.1. Determination of Water and Oil Absorption Capacity

Water absorption capacity (WAC) was determined as described by Rodriguez-Ambriz et al. [24]. A 100 mg protein sample was mixed with 1 mL distilled water and vortexed (Advanced Vortex Mixer—ZX3, VELP Scientifica, Usmate Velate, Italy) for 30 s. The resulting suspension was incubated at room temperature (23 °C) for 30 min and centrifuged for 20 min at 1800× *g* (MPW-251, Med. Instruments, Warsaw, Poland). The supernatant was decanted for 10 min at a 45-degree angle. WAC was calculated by dividing the weight of the absorbed water (g) by the weight of the protein sample (g).

Oil absorption capacity (OAC) was determined by the method of Lin and Zayas [25]. Each protein sample (100 mg) was mixed with 1 mL sunflower oil and vortexed (Advanced Vortex Mixer—ZX3, VELP Scientifica, Usmate (MB), Italy) for 30 s. The mixture was incubated at room temperature (23 °C) for 30 min and subsequently centrifuged at 13,600× *g* for 10 min (MPW-251, Med. Instruments, Warsaw, Poland). The supernatant was decanted and drained for 20 min at a 45-degree angle. OAC was calculated by dividing the weight of the absorbed oil (g) by the weight of the protein sample (g).

The influence of NaCl on WAC and OAC of the protein isolates was evaluated by adding the salt to the test systems to final concentrations of 0.03 or 0.25 M wherever needed.

#### 2.3.2. Emulsifying Properties

Emulsifying activity and emulsion stability were determined as described by Neto et al. [26]. A 5 mL sample solution containing 0.5 mg protein/mL water was homogenized with 5 mL sunflower oil for 60 s at 1000 rpm (Ultra Turrax IKA T18 Basic, Staufen, Germany). The emulsion was centrifuged at 1100× *g* for 5 min, and the height of the emulsified layer was measured. The emulsifying activity was calculated as a ratio of the height of the emulsified layer and the height of the total content of the tube and multiplied by 100 to express in percentage.

Emulsion stability was evaluated after heating at 80 °C. A mixture of 5 mL sample solution (0.5 mg protein/mL water) and 5 mL sunflower oil was homogenized for 60 s at 1000 rpm (Ultra Turrax IKA T18 Basic, Staufen, Germany). The emulsion was heated up to 80 °C in a water bath (WNB 29, Memmert GmbH + Co.KG, Schwabach, Germany) and maintained under the same conditions for 30 min. Subsequently, it was cooled down to room temperature (22 °C) and centrifuged at 1100× *g* for 5 min (MPW-251, Med. Instruments, Warsaw, Poland). Emulsion stability was calculated by the ratio of the height of the emulsified layer after and before heating measured after centrifugation at 1100× *g* for 5 min. NaCl was added to a test system to reach a final concentration of either 0.03 M or 0.25 M as appropriate. The influence of pH on emulsifying properties was tested by varying pH from 2 to 10 with an increment of 2 using NaOH or HCl.

### 2.4. Antioxidant Properties

#### 2.4.1. Determination of Total Flavonoid Content

Total flavonoid contents in PI_2.5–8.5_ or PI_10.5–2.5_ were determined by the aluminium nitrate colorimetric method described by Kivrak et al. [27]. An aliquot of 1 mL protein sample (1% in distilled water, pH 7) was mixed with 0.1 mL 10% aluminium nitrate and 0.1 mL potassium acetate (1 M). An aliquot of 3.8 mL of distilled water (pH 7) was added to the mixture to obtain a total volume of 5 mL. The mixture was vortexed, and the absorbance was measured after 40 min (room temperature) at 415 nm using a spectrophotometer (Spekol 11; Carl Zeiss Jena, Jena, Germany). The total flavonoid content was calculated from a calibration curve constructed with quercetin. The results were obtained as quercetin equivalents (mg) per 100 g dry weight sample and expressed in percentage.

#### 2.4.2. Antioxidant Activity

The antioxidant potential of PI_2.5–8.5_ or PI_10.5–2.5_ to scavenge DPPH radicals was evaluated considering concentrations varying from 0.2% to 1.0% in water (pH 7) with an increment of 0.2. As described by Dimov et al. [28], the analysis was performed with some modifications. Briefly, 0.15 mL isolate sample was mixed with 2.85 mL 0.06 mM DPPH new solution in 96% ethanol. The mixtures were kept at room temperature in darkness, and the absorbance (A) was measured at 517 nm with a spectrophotometer (Spekol 11; Carl Zeiss Jena, Jena, Germany) after 30 min. Antiradical activity (AA) was calculated by using the formula AA = (A_control_ − A_sample_)/A_control_ and multiplied by 100 to express in percentage. The control was treated as the corresponding sample but without adding protein isolates. The synthetic antioxidant butylhydroxytoluene (BHT) was used as a positive control in the same concentrations as for the samples.

Ferric Reducing Antioxidant Power (FRAP) assay was performed as described by Ivanov et al. [29]. Briefly, a 3 mL freshly prepared FRAP reagent [30] was mixed with 0.1 mL 1% PI_2.5–8.5_ or PI_10.5–2.5_ prepared in water (pH 7). The mixture was maintained in the dark for 10 min (37 °C), and the absorbance (A) was measured at 593 nm against blank prepared with water and no sample addition. A standard curve was built with FeSO_4_·7H_2_O. Results were calculated by using the following equation: A = 0.553x − 0.022. The results of FRAP analysis were expressed as μmol Fe^2+^ equivalents per gram dry weight protein isolate [31].

Hydroxyl radical scavenging capacity of PI_2.5–8.5_ or PI_10.5–2.5_ was evaluated by 2-deoxy-D-ribose method as previously described [30]. Briefly, the reaction mixture contained 100 μL of 28 mM 2-deoxy-D-ribose (dissolved in 10 mM KH_2_PO_4_–K_2_HPO_4_ buffer, pH 7.4), 500 μL PI_2.5–8.5_ or PI_10.5–2.5_ water solutions (pH 7) with varying concentrations from 0.2% to 1.0% with an increment of 0.2, 200 μL of 200 μM FeCl_3_ and 1.04 mM ethylenediaminetetraacetic acid (1:1 *v*/*v*) mixed prior to addition, 100 μL of 10 mM H_2_O_2_ and 100 μL of 1 mM ascorbate. The solutions were prepared freshly, and de-aerated water was used for easily oxidizing chemicals. After incubation for 1 h at 37 °C, 1 mL thiobarbituric acid (1% in 50 mM NaOH) and 1 mL trichloroacetic acid (2.8% in water) were added, and the tubes were boiled (100 °C) for 20 min. After cooling, the developed pink chromogen was measured at 532 nm (A) against the appropriate blank (containing only buffer and deoxyribose). Inhibition (in percentage) was calculated by the formula I (%) = 100 − (A_sample_/A_neg.control_) × 100. The negative control contained the reaction mixture with the respective volume of water but not a protein isolate. Mannitol was used as a positive control [32] in the same concentrations as for the samples.

### 2.5. Statistical Evaluation

Presented data are the mean ± standard deviation (SD) of three independent experiments (*n* = 3). Data were analysed by one-way analysis of variance (ANOVA) using Statgraphics Centurion statistical program (version XVI, 2009) (Stat Point Technologies, Ins., Warrenton, VA, USA). Mean differences were established by Fisher’s least significant difference test for paired comparison with a significance level α = 0.05.

## 3. Results and Discussion

### 3.1. Preparation of Protein Isolates

Most plant proteins have isoelectric points in a low acidic region of pH 4.5 to 6 [33]. Rapeseed proteins are unique in having a wide range of isoelectric points [15,34]. According to Lönnerdal and Janson [35], 20% to 40% of the rapeseed proteins had isoelectric points close to pH 11, while the remaining proteins were in the range of pH 4 to 8. The two major rapeseed protein constituents, cruciferin and napin, have the lowest solubility at around pH 7.2 and 10.5, respectively [36]. Thus, the sequential precipitates of the ethanol-treated rapeseed meal protein extract were mixtures of proteins with different isoelectric points, biochemical compositions, and protein profiles as previously evaluated [21]. The authors speculated that the PI_10.5–2.5_, having a precipitation onset at pH 10.5, contained a higher amount of napin and less cruciferin than PI_2.5–8.5_, which explained the better solubility of the PI_10.5–2.5_ in the acidic pH region [21]. The two protein isolates exhibited a solubility behaviour different from that of the protein isolate obtained at a single pH value [37]. The presence of proteins with miscellaneous structures and physicochemical properties as well as phytochemicals with bioactive properties in the PI_2.5–8.5_ or PI_10.5–2.5_ assume multifunctional features of the isolates.

### 3.2. Techno-Functional Properties of PI_2.5–8.5_ and PI_10.5–2.5_

#### 3.2.1. Water- and Oil Absorption Capacities

The PI_10.5–2.5_ exhibited higher water and oil absorption capacities than PI_2.5–8.5_ under all conditions studied (Table 1). WAC and OAC are closely related to protein concentration [38,39], and obtained results were unexpected. The PI_10.5–2.5_ contained a lower amount of crude protein (68.67%) than PI_2.5–8.5_ (72.84%) [21], but the structure, amino acid composition, and conformation of the individual protein molecules mobilized at water/oil should also be considered [40]. According to Ntone et al. [41], napin has a stronger interaction with water/oil molecules than cruciferin due to its specific Janus-like structure, where 45% of hydrophobic amino acids are located at one side of the protein. In contrast, cruciferin, a bigger molecule, has a lower diffusion rate and more even distribution of hydrophobic and hydrophilic domains, leading to a weaker interaction with molecules with different polarities. The addition of NaCl did not influence the WAC of PI_2.5–8.5_ and PI_10.5–2.5_ (Table 1). It might be related to the impurity of both isolates. Ivanova et al. [39] established high sensitivity of WAC of the protein isolate with high protein content (94.25%) to NaCl addition but not of the one with lower protein concentration (75.34%). The WAC of the latter was not modulated by either NaCl supplementation level, 0.03 or 0.25 M. The two levels of NaCl supplementation were chosen as the most employed boundary salt concentrations in food formulation [42].

The ability of proteins to absorb water/oil is an important determinant of texture and mouth feel characteristics of food products [43]. The WAC of PI_2.5–8.5_ and PI_10.5–2.5_ (Table 1) was comparable to the one of other plant protein isolates/concentrates prepared from okra [44], cashew nut [45], and pigeon pea [46]. The OAC of PI_2.5–8.5_ and PI_10.5–2.5_ (Table 1) was higher than that of flours and protein concentrates or isolates from pulses and soybeans [47] and close to the OAC of the protein-rich products obtained from sunflower meal [39], cashew nut [45], and walnut [48].

#### 3.2.2. Emulsifying Properties

Except for pH 6, 8, and 10 (0.25 M NaCl), the overall emulsifying activity of PI_2.5–8.5_ was higher than that of PI_10.5–2.5_ (Table 2). Compared to the emulsifying activity of a rapeseed meal protein isolate prepared by single pH-point precipitation [20], the emulsifying activities of both protein isolates were lower. However, the data demonstrated a weak influence of pH and NaCl supplementation. It is most probably due to the mixed composition of the PI_2.5–8.5_ and PI_10.5–2.5_, which consisted of proteins with different isoelectric points. Thus, the sequential protein precipitates are more advantageous compared to that obtained at single isoelectric points, which exhibit high sensitivity to pH variation, with the lowest emulsifying activity being at the isoelectric point [20,39,45,48]. The low response of the PI_2.5–8.5_ and PI_10.5–2.5_ emulsifying activities to the two factors would allow their application in food systems with a broad pH range and salt concentration. 

**Figure 2:**
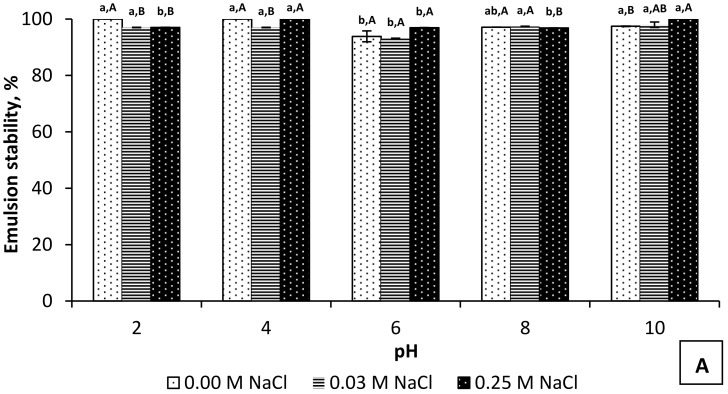

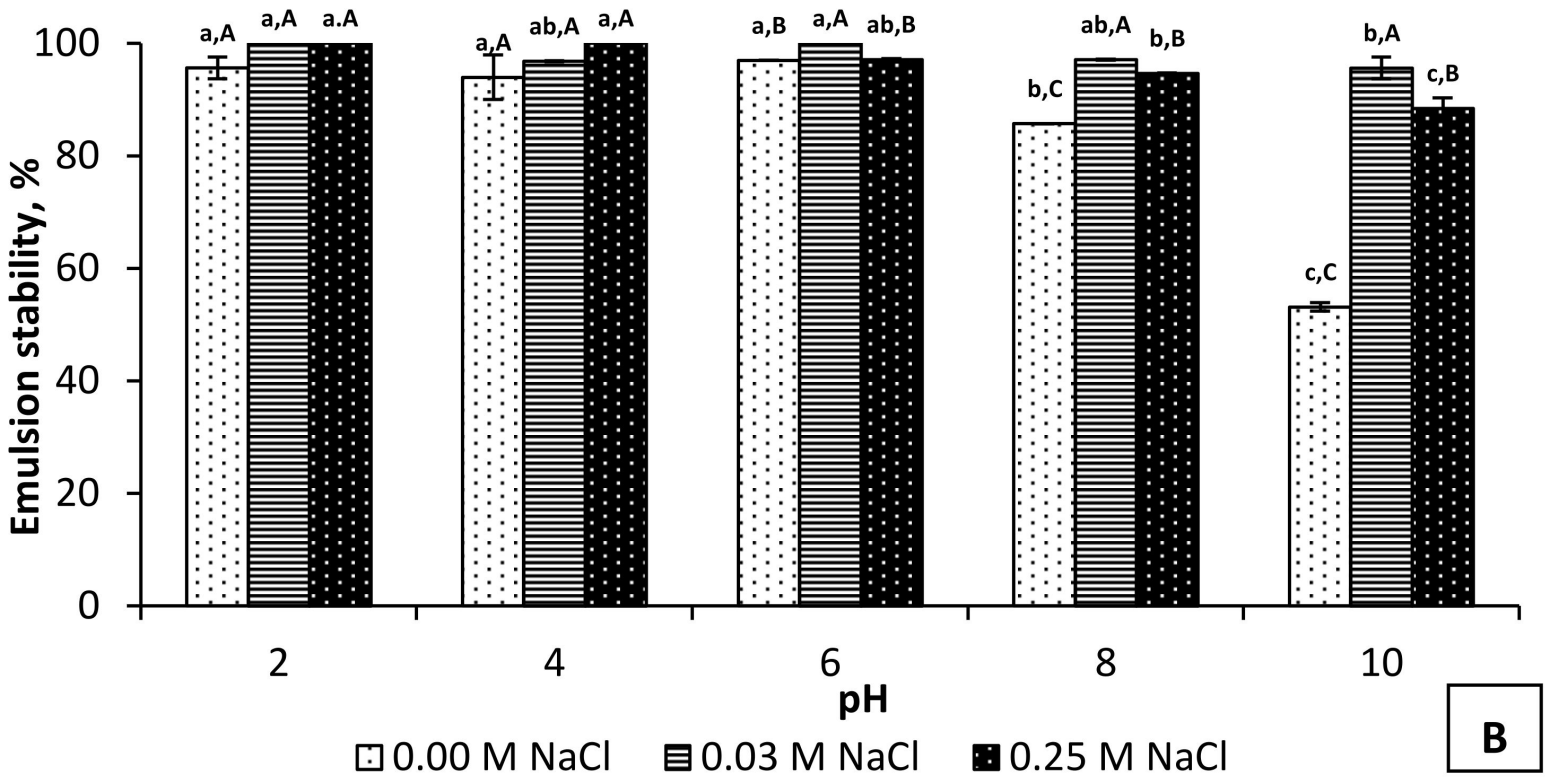
Emulsion stability of PI_2.5–8.5_ (**A**) and PI_10.5–1.5_ (**B**) at different pH and NaCl concentrations. ^a–c^ Means without the same lowercase letter for a particular NaCl concentration differ significantly (*p* < 0.05). ^A–C^ Means without the same capital letter for a particular pH differ significantly (*p* < 0.05).

The PI_2.5–8.5_ and PI_10.5–2.5_ exhibited high emulsion stability 30 min after heating (Figure 2A,B). The emulsion stability of the PI_2.5–8.5_ was either 100% or close to 100% for all pH values studied, except for pH 6 where it reached 93.87% (Figure 2A). This pH is in the range where most rapeseed proteins precipitate [35]. For the PI_10.5–2.5,_ decreases in the emulsion stability were observed at pH 8 (85.71%) and pH 10 (53.15%) (no NaCl addition, Figure 2B). The NaCl supplementation at these pH values enhanced the stability of the emulsions yet was lower than that of the PI_2.5–8.5_ under the same conditions (Figure 2A). With a precipitation onset at pH 10.5, the PI_10.5–2.5_ contained basic proteins having isoelectric points in the pH area from 8 to 10. At the pH close to the isoelectric points, the total charge of proteins is zero. The proteins tend to be more densely packed due to diminished electrostatic repulsive forces and increased hydrophobic interactions, which facilitate droplet flocculation [49]. This is the initial step leading to destabilization of the emulsion and separation of the phases. Far from the isoelectric point, proteins are charged, which facilitates their interaction at the interface and the formation of a stabilizing viscoelastic layer around oil droplets. A recent study by Östbring et al. [50] confirmed the better stabilizing capacity of rapeseed proteins at pH values being far from their isoelectric points. Weak emulsification ability near isoelectric points was reported for sunflower protein isolates [51], potato, rice, and pea protein concentrates [52].

### 3.3. Antioxidant Properties of PI_2.5–8.5_ and PI_10.5–2.5_

Three in vitro methods, DPPH, FRAP, and hydroxyl radical scavenging assay, were used to evaluate the antioxidant properties of PI_2.5–8.5_ and PI_10.5–2.5_ dissolved in water (pH 7). Application of analytical methods differing by their principles is especially important when natural products, containing various bioactive compounds with potentially different antioxidant mechanisms, are evaluated [53]. Considering the influence of the solvent on antioxidant properties of bioactive compounds [54,55], we performed the investigation with the clear understanding that the results might differ from the maximum antioxidant values that could potentially be achieved if other solvents and pH were used. However, water is commonly used in food preparation as a solvation agent, and obtained data would be more significant if applicable under industrial conditions.

**Figure 3:**
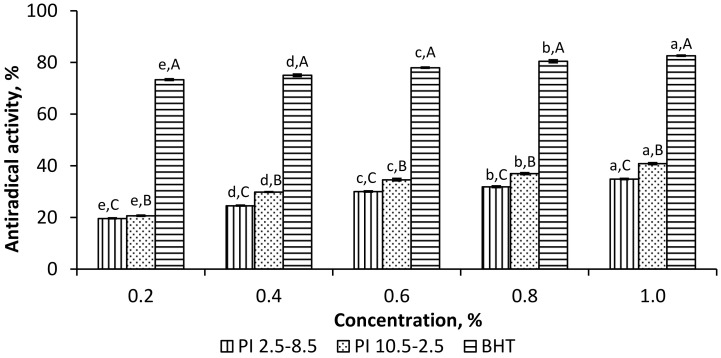
Scavenging effect of ethanol-treated rapeseed meal protein isolates, PI_2.5–8.5_ and PI_10.5–2.5_, on 2,2-diphenyl-1-picryl hydrazyl (DPPH) radical. ^a^^–^^e^ Means of one sample with different concentrations without a common letter differ significantly (*p* < 0.05). ^A^^–^^C^ Means of the samples with the same concentration without a common letter differ significantly (*p* < 0.05).

PI_2.5–8.5_ and PI_10.5–2.5_ demonstrated increasing scavenging potential on DPPH radicals with increasing of their concentrations (Figure 3). In the entire concentration range, from 0.2 to 1.0%, the PI_10.5–2.5_ exhibited a higher antiradical activity than PI_2.5–8.5_. The highest value was achieved at 1.0% PI_10.5–2.5_ (40.83%), approximately half of the antiradical activity of BHT (82.65%) used as a positive control in the study.

**Figure 4:**
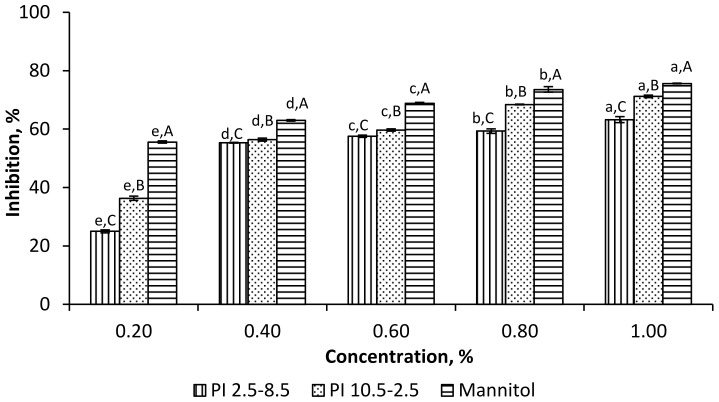
Hydroxyl radical scavenging activity of ethanol-treated rapeseed meal protein isolates, PI_2.5–8.5_ and PI_10.5–2.5_. ^a–e^ Means of one sample with different concentrations without a common letter differ significantly (*p* < 0.05). ^A–C^ Means of the samples with the same concentration without a common letter differ significantly (*p* < 0.05).

A similar trend was observed for the hydroxyl radical scavenging activity of the two protein isolates (Figure 4). For all studied concentrations, the PI_10.5–2.5_ exhibited a higher capacity to neutralize the hydroxyl radicals than PI_2.5–8.5_. It was maximum at 1% PI_10.5–2.5_ (71.25%) and close to the inhibition capacity of mannitol at the same concentration (75.62%). Based on the neutralizing levels and the relative comparison to the compounds used as positive controls, the PI_10.5–2.5_ appeared as a better scavenger of hydroxyl than DPPH radicals.

**Figure 5:**
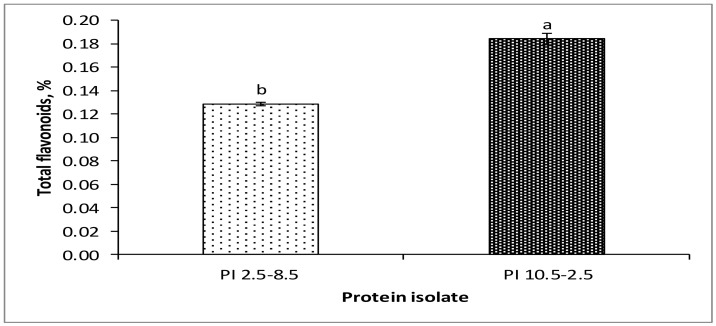
Total flavonoids content of ethanol-treated rapeseed meal protein isolates, PI_2.5–8.5_ and PI_10.5–2.5_. ^a,b^ Means with different superscripts differ significantly (*p* < 0.05).

It was established that PI_10.5–2.5_ contained a higher amount of flavonoids (0.18%) than PI_2.5–8.5_ (0.13%) (Figure 5). Numerous studies, well reviewed by Treml and Smejkal [56], demonstrated flavonoids as potent scavengers of hydroxyl radicals. This feature is structure-related, with some crucial elements, namely ring B hydroxylation, a C2–C3 double bond, a C-4 carbonyl group, and a C-3 hydroxyl group [56]. The hydroxyl radical is one of the most reactive natural free radicals known [56]. This species is highly vulnerable to important biological molecules such as proteins, unsaturated fatty acids, and DNA, as the latter is affected at both primary and secondary structural levels [56]. The two protein isolates, and particularly PI_10.5–2.5_, appeared as valuable products with the potential to decrease oxidative stress caused by hydroxyl radicals.

Concerning total phenols, PI_2.5–8.5_ was the isolate containing a higher amount (0.71%) than PI_10.5–2.5_ (0.42%) [21]. Phenols are a large group of secondary plant metabolites with a high antioxidant capacity [53,57]. Although being tempted to relate the total contents of flavonoids or phenols to obtain antioxidant results, such an interpretation might be inaccurate and misleading. Total phenols have been quantified after ultrasound-assisted extraction with 70% aqueous ethanol solution [21], while the evaluation of the antioxidant capacity was performed after a simple dissolution of the samples in water. Thus, some of the phenols contributing to the total content estimation may not be available for participation in antioxidant reactions of the tests. In addition, the antioxidant properties of the phenols are structure specific, and only the compounds with particular hydroxyl positions in the molecule structure can act as a proton donor [58,59]. da Silva Pereira et al. [60] implied that it was inaccurate to present the antioxidant capacity of a sample as a sum of the antioxidant power of the individual constituents. Phenols and flavonoids tend to bind proteins and form a complex matrix with altered physicochemical properties, including antioxidant ones [61]. Potential synergistic, additive or antagonistic interactions of the single compounds may also influence the total antioxidant potency of natural samples with mixed compositions.

**Figure 6:**
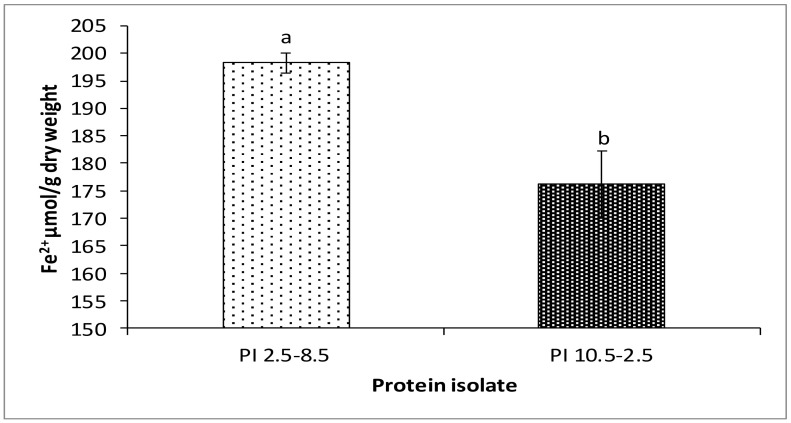
Ferric reducing antioxidant power (FRAP) of ethanol-treated rapeseed meal protein isolates, PI_2.5–8.5_ and PI_10.5–2.5_. ^a,b^ Means with different superscripts differ significantly (*p* < 0.05).

In contrast to DPPH and hydroxyl radical scavenging assays, PI_2.5–8.5_ exhibited a better antioxidant capacity than PI_10.5–2.5_ based on the FRAP measurement approach (Figure 6). This observation might be due to the lower amount of compounds in the PI_10.5–2.5_ able to donate electrons. While the first two methods involve both electron and hydrogen atom transfer reactions, the FRAP method is based only on a single electron transfer reaction, limiting the range of evaluated compounds. Results obtained by FRAP exclude compounds with antioxidant capacity determined by radical quenching (hydrogen atom transfer) such as thiols [59].

Proteins, which are major constituents of PI_2.5–8.5_ and PI_10.5–2.5_, are also excluded from the FRAP evaluation [59]. Proteins/peptides were able to donate protons and neutralize free radicals to terminate the radical chain reactions [62]. Durand et al. [63] reported metal chelating activity of the peptides produced from rapeseed meal proteins with Prolyve (a non-specific microbial endoproteinase with subtilisin activity). Yoshie-Stark et al. [64] demonstrated that pepsin-assisted hydrolysates, obtained from rapeseed protein concentrate, had significant scavenging abilities against DPPH radical. Antioxidant properties of rapeseed protein hydrolysates were also established by others [65,66,67]. While most studies attributed the antioxidant properties to peptides with small molecular weights, Östbring et al. [68] established that non-hydrolysed rapeseed protein precipitates, prepared from five varieties and a mixed blend of cold-pressed rapeseed press cake, were able to reduce lipid oxidation in an emulsion model by using thiobarbituric acid reactive substance assay.

Although rapid, inexpensive, and easy to perform [59], in vitro methods may not provide sufficient estimation of the antioxidant capacity of natural products with a mixed composition such as those of PI_2.5–8.5_ and PI_10.5–2.5_. In vivo methods allow for the evaluation not only of their direct ability to scavenge radicals but also to decrease their production in cells and, therefore, should be considered in a future study to complement current findings [56].

Many foods containing lipids require emulsifiers to prevent two immiscible liquids from separating and to provide good appearance and visual acceptance. Such types of food also require the addition of antioxidants to retard lipid oxidation for better quality and extended shelf-life. Protein–phenol complexes contribute to the formation of emulsions with better physical and oxidative stability [23,69]. During homogenization, proteins are oriented at the water–oil interface and form a visco-elastic layer to prevent the emulsion from flocculation and coalescence, while phenols control the co-oxidation of proteins and lipids. Cong et al. [23] demonstrated that the addition of commonly used emulsifiers, such as kaempferol, phloretin, catechin, resveratrol, and hydroxytyrosol, to sodium caseinate, improved the emulsion’s free-radical scavenging ability, reducing power and the lipid protection effect. Possessing both emulsifying and antioxidant features, the PI_2.5–8.5_ and PI_10.5–2.5_ may belong to the group of natural antioxidant emulsifiers, a term being introduced by McClements and Decker [22]. Since the functional properties of the antioxidant emulsifiers are highly dependent on the type of the polyphenols and/or proteins [23,70,71,72], the compositional complexity of the PI_2.5–8.5_ and PI_10.5–2.5_ impose additional analyses to elucidate their synergistic effect on emulsion stability and lipid oxidation. Still, the current study outlined the two products as potential alternatives to synthetic emulsifiers and antioxidants currently used in the food and nutraceutical industries. Although efficient, synthetic additives have raised safety and consumers’ preferences for natural products with health-promoting effects. Being reasonably soluble in water and with good emulsifying properties, the PI_2.5–8.5_ and PI_10.5–2.5_ would have a broader scope of application as multifunctional ingredients with additional antioxidant properties.

## 4. Conclusions

The study results outlined the PI_2.5–8.5_ and PI_10.5–2.5_ as new natural products with beneficial functional properties. In addition to their fair solubility, the PI_2.5–8.5_ and PI_10.5–2.5_ exhibited good water and oil absorption capacities. The PI_2.5–8.5_ and PI_10.5–2.5_ demonstrated emulsifying properties weakly responding to pH and NaCl modulation, which would allow for their potential application in food systems with a broad pH range and salt concentration. Established antioxidant capacities added value to the protein isolates, which could thus be used as both emulsifiers and antioxidants at a time. The multifunctionality of the protein isolates would contribute to a wider application and, although indirectly, to better and more complete utilization of the rapeseed meal.

## Figures and Tables

**Table 1:** Water and oil absorption capacity of PI_2.5–8.5_ and PI_10.5–1.5_ at different concentrations of NaCl.

Sample	Water Absorption Capacity			Oil Absorption Capacity		
	(g H_2_O/g Sample)			(g oil/g Sample)		
	NaCl Concentration, M			NaCl Concentration, M		
	0	0.03	0.25	0	0.03	0.25
PI_2.5–8.5_	2.11 ± 0.00 ^a,B^	2.25 ± 0.06 ^a,B^	2.23 ± 0.05 ^a,B^	1.68 ± 0.01 ^c,B^	1.73 ± 0.01 ^b,B^	1.90 ± 0.01 ^a,B^
PI_10.5–2.5_	2.36 ± 0.12 ^a,A^	2.42 ± 0.09 ^a,A^	2.68 ± 0.09 ^a,A^	2.03 ± 0.03 ^b,A^	2.05 ± 0.03 ^b,A^	2.36 ± 0.04 ^a,A^

^a–c^ Means in a row for a particular functional property without a common lower case letter differ significantly (*p* < 0.05). ^A,B^ Means in a column without a common capital letter differ significantly (*p* < 0.05).

**Table 2:** Emulsifying activity of PI_2.5–8.5_ and PI_10.5–2.5_ at different pH and NaCl concentrations.

Sample	NaCl Concentration	Emulsifying Activity, %				
		pH				
		2	4	6	8	10
PI_2.5–8.5_	0.00 M	53.85 ± 0.00 ^a,A^	51.17 ± 0.50 ^ab,A^	55.03 ± 1.37 ^b,A^	46.36 ± 0.44 ^ab,B^	52.00 ± 1.88 ^a,A^
	0.03 M	50.38 ± 0.53 ^ab,B^	52.37 ± 1.07 ^a,B^	59.18 ± 0.40 ^a,A^	49.68 ± 1.41 ^a,B^	51.34 ± 0.02 ^a,B^
	0.25 M	47.53 ± 1.48 ^b,A^	48.53 ± 0.06 ^b,A^	45.78 ± 1.00 ^c,A^	45.07 ± 0.00 ^b,A^	46.54 ± 2.07 ^b,A^
PI_10.5–1.5_	0.00 M	48.92 ± 1.53 ^a,AB^	46.51 ± 1.67 ^a,B^	45.85 ± 0.90 ^b,B^	46.67 ± 0.00 ^b,AB^	50.96 ± 0.41 ^a,A^
	0.03 M	49.28 ± 0.00 ^a,AB^	48.09 ± 0.56 ^a,AB^	50.70 ± 0.98 ^a,A^	46.95 ± 1.41 ^b,B^	46.25 ± 0.59 ^b,B^
	0.25 M	44.68 ± 0.55 ^b,D^	46.77 ± 1.50 ^a,CD^	48.29 ± 0.40 ^ab,BC^	51.02 ± 0.47 ^a,AB^	53.44 ± 1.04 ^a,A^

^a–c^ Means in a column for a sample without a common lower case letter differ significantly (*p* < 0.05). ^A–D^ Means in a row without a common capital letter differ significantly (*p* < 0.05).

## Data Availability

Data are available on request.

## Sample Availability

Samples of the compounds are available from the authors.

## References

[1] Raboanatahiry N., Li H., Yu L., Li M. (2021). Rapeseed (*Brassica napus*): Processing, Utilization, and Genetic Improvement. Agronomy.

[2] Piazza G.J., Foglia T.A. (2001). Rapeseed oil for oleochemical usage. Eur. J. Lipid Sci. Technol..

[3] Aukema H., Campbell L., Daun J.K., Michael E.N.A., Hickling D. (2011). Oil nutrition and utilization. Canola: Chemistry, Production, Processing, and Utilization.

[4] Senthilselvan A., Zhang Y., Dosman J.A., Barber E.M., Holfeld L.E., Kirychuk S.P., Cormier Y., Hurst T.S., Rhodes C.S. (1997). Positive Human Health Effects of Dust Suppression with Canola Oil in Swine Barns. Am. J. Respir. Crit. Care Med..

[5] Jaastad G. (2007). Late dormant rapeseed oil treatment against black cherry aphid and cherry fruit moth in sweet cherries. J. Appl. Entomol..

[6] Heuzé V., Tran G., Sauvant D., Lessire M., Lebas F. (2020). Rapeseed Meal. Feedipedia, a Programme by INRAE, CIRAD, AFZ and FAO. https://www.feedipedia.org/node/52.

[7] USDA, United States Department of Agriculture Foreign Agricultural Service, Oilseeds and Products Annual, EU Oilseeds Report Annual 2020, Global Agricultural Information Network, Vienna 15 April 2021. https://www.fas.usda.gov/data/european-union-oilseeds-and-products-annual-1.

[8] Fu Z., Su G., Yang H., Sun Q., Zhong T., Wang Z. (2021). Effects of Dietary Rapeseed Meal on Growth Performance, Carcass Traits, Serum Parameters, and Intestinal Development of Geese. Animals.

[9] Le D.T., Chu H.D., Le N.Q. (2016). Improving Nutritional Quality of Plant Proteins Through Genetic Engineering. Curr. Genom..

[10] Nega T. (2018). Review on Nutritional Limitations and Opportunities of using Rapeseed Meal and other Rape Seed by—Products in Animal Feeding. J. Nutr. Health Food Eng..

[11] Henchion M., Hayes M., Mullen A.M., Fenelon M., Tiwari B. (2017). Future Protein Supply and Demand: Strategies and Factors Influencing a Sustainable Equilibrium. Foods.

[12] Tilman D., Clark M. (2014). Global diets link environmental sustainability and human health. Nature.

[13] Scarborough P., Appleby P.N., Mizdrak A., Briggs A., Travis R.C., Bradbury K., Key T.J. (2014). Dietary greenhouse gas emissions of meat-eaters, fish-eaters, vegetarians and vegans in the UK. Clim. Chang..

[14] van der Spiegel M., Noordam M., van der Fels-Klerx H. (2013). Safety of Novel Protein Sources (Insects, Microalgae, Seaweed, Duckweed, and Rapeseed) and Legislative Aspects for Their Application in Food and Feed Production. Compr. Rev. Food Sci. Food Saf..

[15] Tan S.H., Mailer R.J., Blanchard C.L., Agboola S.O. (2011). Canola Proteins for Human Consumption: Extraction, Profile, and Functional Properties. J. Food Sci..

[16] Wanasundara J.P., McIntosh T.C., Perera S.P., Withana-Gamage T.S., Mitra P. (2016). Canola/rapeseed protein-functionality and nutrition. OCL.

[17] Pedroche J., Yust M.D.M., Lqari H., Giron-Calle J., Alaiz M., Vioque J., Millán F. (2004). Brassica carinata protein isolates: Chemical composition, protein characterization and improvement of functional properties by protein hydrolysis. Food Chem..

[18] Yoshie-Stark Y., Wada Y., Wäsche A. (2008). Chemical composition, functional properties, and bioactivities of rapeseed protein isolates. Food Chem..

[19] Das Purkayastha M., Gogoi J., Kalita D., Chattopadhyay P., Nakhuru K.S., Goyary D., Mahanta C.L. (2014). Physicochemical and Functional Properties of Rapeseed Protein Isolate: Influence of Antinutrient Removal with Acidified Organic Solvents from Rapeseed Meal. J. Agric. Food Chem..

[20] Kalaydzkiev H., Ivanova P., Silva C., Chalova V.I. (2019). Functional Properties of Protein Isolate and Acid Soluble Protein-Rich Ingredient Co-Produced from Ethanol-Treated Industrial Rapeseed Meal. Pol. J. Food Nutr. Sci..

[21] Kalaydzhiev H., Georgiev R., Ivanova P., Stoyanova M., Silva C.L.M., Chalova V.I. (2020). Enhanced Solubility of Rapeseed Meal Protein Isolates Prepared by Sequential Isoelectric Precipitation. Foods.

[22] McClements D.J., Decker E. (2017). Interfacial Antioxidants: A Review of Natural and Synthetic Emulsifiers and Coemulsifiers That Can Inhibit Lipid Oxidation. J. Agric. Food Chem..

[23] Gong T., Tian D., Hu C.Y., Guo Y.R., Meng Y.H. (2021). Improving antioxidant ability of functional emulsifiers by conjugating polyphenols to sodium caseinate. LWT.

[24] Rodríguez-Ambriz S.L., Martínez-Ayala A.L., Millán F., Dávila-Ortíz G. (2005). Composition and Functional Properties of Lupinus campestris Protein Isolates. Mater. Veg..

[25] Lin C.S., Zayas J.F. (1987). Functionality of Defatted Corn Germ Proteins in a Model System: Fat Binding Capacity and Water Retention. J. Food Sci..

[26] Neto V.Q., Narain N., Silva J.B., Bora P.S. (2001). Functional properties of raw and heat processed cashew nut (Anacardium occidentale, L.) kernel protein isolates. Food/Nahr..

[27] Kivrak Ş., Göktürk T., Kivrak I., Kaya E., Karababa E. (2019). Investigation of phenolic profiles and antioxidant activities of some Salvia species commonly grown in Southwest Anatolia using UPLC-ESI-MS/MS. Food Sci. Technol..

[28] Dimov I., Petkova N., Nakov G., Taneva I., Ivanov I., Stamatovska V. (2018). Improvement of antioxidant potential of wheat flours and breads by addition of medicinal plants. Ukr. Food J..

[29] Ivanov I., Vrancheva R.Z., Marchev A.S., Petkova N.T., Aneva I.Y., Denev P.P., Georgieva V.G., Pavlov A.I. (2014). Antioxidant activities and phenolic compounds in Bulgarian Fumaria species. Int. J. Curr. Microbiol. Appl. Sci..

[30] Georgiev R., Ivanov I.G., Ivanova P., Tumbarski Y., Kalaydzhiev H., Dincheva I.N., Badjakov I.K., Chalova V.I. (2021). Phytochemical Profile and Bioactivity of Industrial Rapeseed Meal Ethanol-Wash Solutes. Waste Biomass-Valorization.

[31] Irshad M., Zafaryab M., Singh M., Rizvi M. (2012). Comparative Analysis of the Antioxidant Activity of Cassia fistula Extracts. Int. J. Med. Chem..

[32] Yan X., Nagata T., Fan X. (1998). Antioxidative activities in some common seaweeds. Mater. Veg..

[33] Sim S.Y.J., Srv A., Chiang J.H., Henry C.J. (2021). Plant Proteins for Future Foods: A Roadmap. Foods.

[34] Tan S.H., Mailer R.J., Blanchard C.L., Agboola S.O. (2011). Extraction and characterization of protein fractions from Australian canola meals. Food Res. Int..

[35] Lönnerdal B., Janson J.-C. (1972). Studies on Brassica seed proteins: I. The low molecular weight proteins in rapeseed. Isolation and characterization. Biochim. et Biophys. Acta (BBA)—Protein Struct..

[36] Wanasundara J.P.D. (2011). Proteins ofBrassicaceaeOilseeds and their Potential as a Plant Protein Source. Crit. Rev. Food Sci. Nutr..

[37] Ivanova P., Kalaydzhiev H., Rustad T., Silva C.L.M., Chalova V.I. (2017). Comparative biochemical profile of protein-rich products obtained from industrial rapeseed meal. Emir. J. Food Agric..

[38] Zayas J.F. (1997). Water Holding Capacity of Proteins. Functionality of Proteins in Food.

[39] (2014). Functional properties of proteins isolated from industrially produced sunflower meal. Int. J. Food Stud..

[40] Kneifel W., Paquin P., Abert T., Richard J.-P. (1991). Water-Holding Capacity of Proteins with Special Regard to Milk Proteins and Methodological Aspects—A Review. J. Dairy Sci..

[41] Ntone E., van Wesel T., Sagis L.M., Meinders M., Bitter J.H., Nikiforidis C.V. (2020). Adsorption of rapeseed proteins at oil/water interfaces. Janus-like napins dominate the interface. J. Colloid Interface Sci..

[42] Dragoev S.G., Vulkova-Yorgova K.I., Balev D.K. (2008). Technology of Functional and Special Meat and Fish Products.

[43] Okezie B.O., Bello A. (1988). Physicochemical and Functional Properties of Winged Bean Flour and Isolate Compared with Soy Isolate. J. Food Sci..

[44] Nnamezie A.A., Famuwagun A.A., Gbadamosi S.O. (2021). Characterization of okra seed flours, protein concentrate, protein isolate and enzymatic hydrolysates. Food Prod. Process. Nutr..

[45] Ogunwolu S.O., Henshaw F.O., Mock H.-P., Santros A., Awonorin S.O. (2009). Functional properties of protein concentrates and isolates produced from cashew (Anacardium occidentale L.) nut. Food Chem..

[46] Aa O. (2017). Emulsification and Foaming Properties of Locust Bean (Parkiabiglobosa) and Pigeon Pea (Cajanuscajan) Seed Flours and their Protein Isolates. Nutr. Food Sci. Int. J..

[47] Wang N., Maximiuk L., Fenn D., Nickerson M.T., Hou A. (2020). Development of a method for determining oil absorption capacity in pulse flours and protein materials. Cereal Chem..

[48] Mao X., Hua Y. (2012). Composition, Structure and Functional Properties of Protein Concentrates and Isolates Produced from Walnut (*Juglans regia* L.). Int. J. Mol. Sci..

[49] McClements D.J. (2004). Protein-stabilized emulsions. Curr. Opin. Colloid Interface Sci..

[50] Östbring K., Matos M., Marefati A., Ahlström C., Gutiérrez G. (2021). The Effect of pH and Storage Temperature on the Stability of Emulsions Stabilized by Rapeseed Proteins. Foods.

[51] Malik M.A., Saini C.S. (2018). Improvement of functional properties of sunflower protein isolates near isoelectric point: Application of heat treatment. LWT.

[52] da Silva A.M.M., Almeida F.S., Sato A.C.K. (2020). Functional characterization of commercial plant proteins and their application on stabilization of emulsions. J. Food Eng..

[53] Kebede M., Admassu S. (2019). Application of Antioxidants in Food Processing Industry: Options to Improve the Extraction Yields and Market Value of Natural Products. Adv. Food Technol. Nutr. Sci.—Open J..

[54] Zhou K., Yu L. (2004). Effects of extraction solvent on wheat bran antioxidant activity estimation. LWT.

[55] Kerchev P., Ivanov S. (2008). Influence of Extraction Techniques and Solvents on the Antioxidant Capacity of Plant Material. Biotechnol. Biotechnol. Equip..

[56] Treml J., Šmejkal K. (2016). Flavonoids as Potent Scavengers of Hydroxyl Radicals. Compr. Rev. Food Sci. Food Saf..

[57] Selamoglu Z., Sevindik M., Bal C., Ozaltun B., Sen I., Pasdaran A. (2020). Antioxidant, antimicrobial and DNA protection activities of phenolic content of *Tricholoma virgatum* (Fr.) P.Kumm. Biointerface Res. Appl. Chem..

[58] Rice-Evans C.A., Miller N.J., Paganga G. (1996). Structure-antioxidant activity relationships of flavonoids and phenolic acids. Free Radic. Biol. Med..

[59] Lewoyehu M., Amare M. (2019). Comparative evaluation of analytical methods for determining the antioxidant activities of honey: A review. Cogent Food Agric..

[60] Pereira A.C.D.S., Wurlitzer N., Dionisio A.P., Soares M.V.L., Bastos M.D.S.R., Alves R.E., Brasil I.M. (2015). Synergistic, additive and antagonistic effects of fruit mixtures on total antioxidant capacities and bioactive compounds in tropical fruit juices. Arch. Latinoam. de Nutr..

[61] Arts M.J.T.J., Haenen G.R.M.M., Wilms L.C., Beetstra S.A.J.N., Heijnen C.G.M., Voss A.H.-P., Bast A. (2002). Interactions between Flavonoids and Proteins: Effect on the Total Antioxidant Capacity. J. Agric. Food Chem..

[62] Wang L., Yang J., Wang Y., Zhang J., Gao Y., Yuan J., Su A., Ju X. (2016). Study on Antioxidant Activity and Amino Acid Analysis of Rapeseed Protein Hydrolysates. Int. J. Food Prop..

[63] Durand E., Beaubier S., Fine F., Villeneuve P., Kapel R. (2021). High Metal Chelating Properties from Rapeseed Meal Proteins to Counteract Lipid Oxidation in Foods: Controlled Proteolysis and Characterization. Eur. J. Lipid Sci. Technol..

[64] Yoshie-Stark Y., Wada Y., Schott M., Wäsche A. (2006). Functional and bioactive properties of rapeseed protein concentrates and sensory analysis of food application with rapeseed protein concentrates. LWT.

[65] He R., Girgih A.T., Malomo S.A., Ju X., Aluko R.E. (2013). Antioxidant activities of enzymatic rapeseed protein hydrolysates and the membrane ultrafiltration fractions. J. Funct. Foods.

[66] Mäkinen S., Johannson T., Gerd E.V., Pihlava J.M., Pihlanto A. (2012). Angiotensin I-converting enzyme inhibitory and antioxidant properties of rapeseed hydrolysates. J. Funct. Foods.

[67] Pan M., Jiang T.S., Pan J.L. (2009). Antioxidant Activities of Rapeseed Protein Hydrolysates. Food Bioprocess Technol..

[68] Östbring K., Tullberg C., Burri S., Malmqvist E., Rayner M. (2019). Protein Recovery from Rapeseed Press Cake: Varietal and Processing Condition Effects on Yield, Emulsifying Capacity and Antioxidant Activity of the Protein Rich Extract. Foods.

[69] Djuardi A.U.P., Yuliana N.D., Ogawa M., Akazawa T., Suhartono M.T. (2020). Emulsifying properties and antioxidant activity of soy protein isolate conjugated with tea polyphenol extracts. J. Food Sci. Technol..

[70] Sun J., Jing H., Mu Y., McClements D.J., Dong S., Xu B. (2020). Fabrication of antioxidant emulsifiers from natural ingredients: Conjugation of egg white proteins with catechin and chlorogenic acid. Food Hydrocoll..

[71] Quan T.H., Benjakul S., Sae-Leaw T., Balange A.K., Maqsood S. (2019). Protein–polyphenol conjugates: Antioxidant property, functionalities and their applications. Trends Food Sci. Technol..

[72] Pham L.B., Wang B., Zisu B., Adhikari B. (2019). Complexation between flaxseed protein isolate and phenolic compounds: Effects on interfacial, emulsifying and antioxidant properties of emulsions. Food Hydrocoll..

